# Insula and inferior frontal triangularis activations distinguish between conditioned brain responses using emotional sounds for basic BCI communication

**DOI:** 10.3389/fnbeh.2014.00247

**Published:** 2014-07-21

**Authors:** Linda van der Heiden, Giulia Liberati, Ranganatha Sitaram, Sunjung Kim, Piotr Jaśkowski, Antonino Raffone, Marta Olivetti Belardinelli, Niels Birbaumer, Ralf Veit

**Affiliations:** ^1^Department of Cognitive Psychology, University of Finance and ManagementPawia, Warsaw, Poland; ^2^Institute of Medical Psychology and Behavioral Neurobiology, Eberhard Karls-UniversityTübingen, Germany; ^3^Interuniversity Centre for Research on Cognitive Processing in Natural and Artificial Systems (ECONA)Rome, Italy; ^4^Institute of Neuroscience, Université Catholique de LouvainBrussels, Louvain-la-Neuve, Belgium; ^5^Department of Biomedical Engineering, University of FloridaGainesville, FL, USA; ^6^Biomedical Engineering, Sri Chitra Tirunal Institute of Medical Sciences and TechnologyTrivandrum, India; ^7^Department of Psychology, University “Sapienza” of RomeRome, Italy; ^8^Ospedale San Camillo—IRCCS, Istituto di Ricovero e Cura a Carattere ScientificoVenezia Lido, Italy

**Keywords:** classical conditioning, emotions, fMRI, Insula, inferior frontal triangularis, BCI

## Abstract

In order to enable communication through a brain-computer interface (BCI), it is necessary to discriminate between distinct brain responses. As a first step, we probed the possibility to discriminate between affirmative (“yes”) and negative (“no”) responses using a semantic classical conditioning paradigm, within an fMRI setting. Subjects were presented with congruent and incongruent word-pairs as conditioned stimuli (CS), respectively eliciting affirmative and negative responses. Incongruent word-pairs were associated to an unpleasant unconditioned stimulus (scream, US1) and congruent word-pairs were associated to a pleasant unconditioned stimulus (baby-laughter, US2), in order to elicit emotional conditioned responses (CR). The aim was to discriminate between affirmative and negative responses, enabled by their association with the positive and negative affective stimuli. In the late acquisition phase, when the US were not present anymore, there was a strong significant differential activation for incongruent and congruent word-pairs in a cluster comprising the left insula and the inferior frontal triangularis. This association was not found in the habituation phase. These results suggest that the difference in affirmative and negative brain responses was established as an effect of conditioning, allowing to further investigate the possibility of using this paradigm for a binary choice BCI.

## Introduction

The possibility to produce two differentiable conditioned responses, corresponding to “affirmative” and “negative” thinking, could be exploited for basic yes/no communication through a brain-computer interface (BCI). This would allow individuals who are not able to use standard communication pathways (e.g., because of severe motor disability or expressive deficits) to convey information on their basic needs. BCIs are traditionally based on operant conditioning (Wolpaw et al., 2002), which may however be problematic for some users, such as completely locked-in state individuals (Birbaumer, 2006) or persons with dementia (Liberati et al., 2012a).

In the present study, we investigated the possibility to use classical conditioning to discriminate between different brain responses. The advantages of such paradigm shift is that, since classical conditioning does not require users to perform cognitively challenging tasks, it could be used for communicating with cognitively impaired patients (e.g., with dementia Liberati et al., 2012a,b).

Differential classical conditioning has been well studied and described over the last century. The basic principle of this learning mechanism is that a conditioned stimulus (CS+) is paired with an unconditioned stimulus (US), while another CS remains unpaired (CS−). The pairing of CS with US results in a conditioned response (CR; Pavlov, 1927) reflecting a new learned stimulus-response association. Many variations of this procedure have been applied, using different CS modalities and pleasant/appetitive or unpleasant/aversive US. Semantic classical conditioning refers to the conditioning of responses to meaningful words or sentences, irrespective of the specific letters or sounds that constitute the words (Razran, 1939, 1961). The repeated association of words or sentences with a significant US, such as a painful stimulus, produces a CR, i.e., measured at the level of cortical evoked responses (Montoya et al., 1996). Recently, semantic conditioning experiments have been performed while electroencephalographic (EEG) potentials were measured (De Massari et al., 2012; Furdea et al., 2012; Ruf et al., 2013) using two different aversive auditory US or a single aversive electrical shock US. Up to now, semantic conditioning has not been investigated using functional magnetic resonance imaging (fMRI).

In the present fMRI study, we introduced a semantic double conditioning paradigm to condition two different responses, an affirmative and a negative one, in view of developing a BCI to allow basic yes/no communication. For this purpose, an innovative approach was tested in which congruent and incongruent word-pairs, eliciting respectively affirmative and negative thinking, were associated with two different emotional sounds, a pleasant and an unpleasant one.

Our aim was to assess whether it is possible to discriminate between the congruent and incongruent word-pairs, using a semantic conditioning protocol to condition two distinct brain responses at the same time, by associating two emotional stimuli to word-pairs presented aurally. We hypothesized that, after classical conditioning, the semantic stimuli would elicit differentially conditioned responses in emotion-related brain areas. More specifically, we expected that word-pairs associated with unpleasant emotional stimuli would elicit a negative emotional response (e.g., associated with ACC, insula and amygdala activation) (Büchel et al., 1998, 1999) and word-pairs associated with pleasant emotional stimuli a positive emotional response (e.g., associated with amygdala, hippocampus and prefrontal cortex activation) (Ito et al., 2005; Costa et al., 2010). In other words, we addressed two challenges, namely double semantic auditory conditioning using emotional sounds and—through this approach—the elicitation of distinctive brain responses, to enable basic yes/no BCI communication.

## Materials and methods

### Participants

Ten right-handed, native German speaking, healthy individuals (5 males, 5 females), ranging in age from 21 to 28 (mean age = 25.3, *SD* = 1.77 years), participated in this study. All participants gave written informed consent prior to participation in the fMRI experiment. The study was approved by the Ethics Committee of the Medical Faculty of the University of Tübingen and was performed in compliance with the Code of Ethics of the World Medical Association (Declaration of Helsinki).

### Stimuli

The stimuli consisted of 300 German word-pairs, read aloud by a native German speaker, recorded using a SpeedLink USB microphone and QuickTime Player 7 program for Macintosh.

Each word-pair included a superordinate category (e.g., “animals”) and a subordinate object (e.g., “dog”). Half of the word-pairs were congruent (e.g., “Obst-Apfel,” “Fruit-Apple”), half incongruent (e.g., “Obst-Hund,” “Fruit-Dog”). Congruence was given by the belonging of the object to the category. The categories that were used were Animals, Countries, Fruit, Furniture, Sports, Clothing, Instruments, Drinks, Crockery, and Jobs. The word-pairs were short (1.5 s) and simple, so that they did not represent a cognitive challenge. Word-pairs were chosen, instead of questions or sentences, as they could be more easily standardized in length, and could be also understandable by individuals with some degree of cognitive deficit. In fact, different studies have shown that even in presence of cognitive impairment, basic semantic information that may not be explicitly accessible could be relatively intact at the implicit level (Nebes, 1994; Laisney et al., 2011).

The brain responses associated to the congruent and incongruent word-pairs (associated respectively to “yes” and “no” thinking) constituted the conditioned stimuli (CS). The unconditioned stimuli (US) were two standardized emotional sounds drawn from the International Affective Digitized Sounds (IADS, Bradley and Lang, 1999; Stevenson and James, 2008): a pleasant emotional stimulus (a baby-laughter) and an unpleasant emotional stimulus (a scream). The duration of each US was also 1.5 s. To ascertain that all stimuli had the same precise length, their duration was adjusted using the software program Audacity 1.3.14 Beta for Mac OS X. As our final aim was communication, congruent word-pairs (eliciting affirmative thinking) where always associated with the pleasant emotional stimulus, and incongruent word-pairs (eliciting negative thinking) were always associated with the unpleasant emotional stimulus, in order to maximize the strength of the rewarding/aversive effects.

Stimuli presentation in the fMRI scanner was performed with a software interface developed in Matlab v. 6.5 (Mathworks, Inc., Sherbon, MA). Participants heard all auditory stimuli through MRI-compatible headphones with efficient gradient noise suppression (up to 45 dB) and a filter system with more than 90 dB RF-suppression (MR confon System, Leibniz-Institute for Neurobiology at Magdeburg, Germany).

### Experimental paradigm

The paradigm consisted of a single session divided into six blocks (Table 1). In the first block, defined as habituation phase, 25 incongruent word-pairs (CS1), 25 congruent word-pairs (CS2), 25 unpleasant emotional stimuli (scream, US1) and 25 pleasant emotional stimuli (laughter, US2) were presented to the subject in a pseudo-random order, so that the same stimulus was never presented consecutively more than three times. The inter-stimulus interval (ISI) was also randomized to optimize design efficiency, and could last for 6, 7.5, or 9 s. The habituation phase served to evaluate the activations relative to each type of stimulus individually, before their association in the conditioning process. In the second and third blocks, which were structured identically and constituted the early acquisition phase, 25 incongruent word-pairs (CS1) and 25 congruent word-pairs (CS2) were presented in a pseudo-random order, such that the same condition (congruent or incongruent) was never presented consecutively more than three times. Each word-pair was immediately followed by an US: scream (US1) after incongruent word-pairs and laughter (US2) after congruent word-pairs. Also in the fourth and fifth block (late acquisition phase), 25 CS1 and 25 CS2 were presented in a pseudo-random order. In the fourth block, only 10 CS1 and 10 CS2 were paired (40% of the word-pairs were followed by the appropriate US) with US1 and US2, respectively. In the fifth block, only 5 CS1 and 5 CS2 were paired (20%). In the sixth and final block (extinction block), none of the 50 word-pairs, also presented in a pseudo-random order, were followed by an US. The gradual diminution of the percentage of word-pairs followed by emotional stimulation served to verify whether the conditioning had taken place, meaning that congruent and incongruent word-pairs could be discriminated thanks to their association to the emotional sounds, even when the emotional sounds were not present anymore.

**Table 1:** **Summary of the protocol**.

**Block**	**Phase**	**CS1 + US1**	**CS1**	**CS2 + US2**	**CS2**	**US1**	**CS2**
1	Habituation		25		25	25	25
2	Early acquisition	25		25			
3		25		25			
4	Late acquisition	10	15	10	15		
5		5	20	5	20		
6	Extinction		25		25		

*The classical conditioning procedure comprised six blocks: one habituation block (Wolpaw et al., 2002), two early acquisition blocks (Birbaumer, 2006; Liberati et al., 2012a), two late acquisition blocks (Pavlov, 1927; Liberati et al., 2012b), and one extinction block (Razran, 1939). In the habituation, incongruent word-pairs (CS1), congruent word-pairs (CS2), the scream (US1) and the laughter (US2) were all presented individually (25 times each). In the early acquisition, CS1 was always paired with US1 (25 times) and CS2 was always paired with US2 (25 times). In the late acquisition, the number of CS paired with US progressively decreased. In the extinction, only CS1 (25 times) and CS2 (25 times) were presented, without US*.

### Behavioral measures

Participants were instructed to use the Self-Assessment Manikin (SAM, Bradley and Lang, 1994) to rate the valence (pleasantness/unpleasantness) and the arousal related to the two emotional US (scream and laughter), both at the end of the first block and at the end of the fifth block, to allow for the comparison of the two stimuli and to assess whether the subjects habituated to them throughout the measurement. The SAM comprises two 9–point scales, ranging from “pleasant” to “unpleasant” and from “not arousing at all” to “very arousing,” respectively.

### fMRI data acquisition

The experiment was performed using a Siemens AG (Erlangen, Germany) 3T Trio MRI scanner. Functional T2^*^-weighted images were acquired with a standard 12-channels head coil, in transversal orientation (*TR* = 1.5 s, *TE* = 30 ms, flip angle = 70°, matrix 64 × 64, voxel size = 3.3 × 3.3 × 5.0 mm^3^, 16 slices, 1 mm gap, bandwidth = 1.954 kHz/pixel) covering the whole brain. Moreover, a high-resolution T1-weighted anatomical scan of the whole brain was acquired from each participant (MPRAGE, matrix size = 256 × 256, 160 slices, 1 mmisotropic voxels, *TR* = 2300 ms., *TE* = 3.93 ms., *TI* = 1100 ms, flip angle α = 8°). The first 10 volumes of every block were discarded to permit T1 equilibrium.

### fMRI data analysis

Data were analyzed using Statistical Parametric Mapping (SPM8, Wellcome Department of Imaging Neuroscience, London, UK) run on Matlab R2008b (Mathworks, Inc., Sherborn, MA, USA). Images of each subject were realigned and unwarped to correct for head movement, and were normalized to a standard Echo-Planar Imaging (EPI) template in Montreal Neurological Institute (MNI) space. Spatial smoothing was applied using a Gaussian kernel with full width at half-maximum of 9 mm. Prior to statistical analyses data were high-pass filtered (cutoff 128 s) and low-pass filtered (AR, Wolpaw et al., 2002).

Statistical analysis was carried out using the general linear model (GLM) with the canonical hemodynamic response function (HRF) as a basis set. In a first level analysis, regressors were defined to discriminate between paired and unpaired word-pairs separately for each block, condition and conditioning phase. The regressors were CS1 paired, CS2 paired, CS1 unpaired, CS2 unpaired, US1 and US2. The six movement regressors for each block were included as confounds in the design matrix to capture residual movement-related variance. The following contrasts were defined on the first level: US1 vs. US2, and unpaired CS1 compared to CS2 in the habituation phase CS1 paired, CS2 paired in the early acquisition phase and CS1 unpaired vs. CS2 unpaired, in the late acquisition phase as well as during extinction. Moreover contrasts were defined combining paired and unpaired word-pairs in the early and late acquisition. In a second level analysis, corresponding contrast images of all subjects were used to assess the main effects of conditioning. A paired *t*-test, performed by including the individual contrast images for US1 and US2 during habituation, was computed in order to detect significantly activated brain regions related to these emotional unconditioned stimuli during habituation. A paired *t*-tests was also performed to detect differences in brain regions involved in the laughter vs. scream and congruent vs. incongruent word-pairs in the extinction phases. To investigate the difference in brain regions for congruent and incongruent word-pairs, we performed a 2 × 2 full-factorial model with the factors Phase (early acquisition and late acquisition) and Condition (congruent and incongruent). Moreover, two different models were computed to observe activations in the early acquisition and late acquisition individually, to evaluate the progression of the conditioning process. For all group statistics a cluster-level threshold of *p* < 0.05 corrected for the Family Wise Error rate (FWER) of the whole brain was used.

## Results

### Behavioral data

#### SAM ratings

A two-sample *t*-test indicated that participants rated the scream as significantly more unpleasant [block 1: *t*_(20)_ = 10.62, block 5: *t*_(20)_ = 3.09, *p* < 0.01] and more arousing [block 1: *t*_(20)_ = 5.87, block 5: *t*_(20)_ = 2.66, *p* < 0.02] than the laughter, both at the beginning and at the end of the measurement. The arousal associated to the scream was significantly less at the end of block 5 compared to block 1 [*t*_(20)_ = 2.96, *p* < 0.01], although no significant difference was found for the laughter (p = 0.3). The valence associated to the scream and laughter did not change significantly during the experiment (*p* = 0.2 in both cases).

### Neuroimaging data

In the habituation phase (Table 2), significant differential activations were only found for the scream vs. laughter (US1>US2) contrast, in the left middle cingulate gyrus (MCG), in the right inferior frontal triangularis (IFT), and in the superior frontal gyrus (SFG). No difference was found for the reverse contrast (US2>US1), nor for the incongruent vs. congruent (CS1>CS2) and congruent vs. incongruent (CS2>CS1) contrasts.

**Table 2:** **Habituation**.

**Contrast**	**Region (Brodmann's Area)**		***t*-value**	**MNI coordinate**		
				***x***	***y***	***z***
Scream > Laughter	Middle cingulate gyrus (BA 24)	L	6.03	−18	−19	43
	Inferior frontal triangularis	R	5.56	54	20	4
	Superior frontal gyrus	R	5.25	24	47	19
	Superior frontal gyrus (BA 9)	R	5.25	15	53	40
Laughter > Scream	No differential activations					
Incongruent > Congruent without US	No differential activations					
Congruent > Incongruent without US	No differential activations					

*P < 0.05 FWE corrected on cluster level; R, Right; L, Left*.

**Figure 1:**
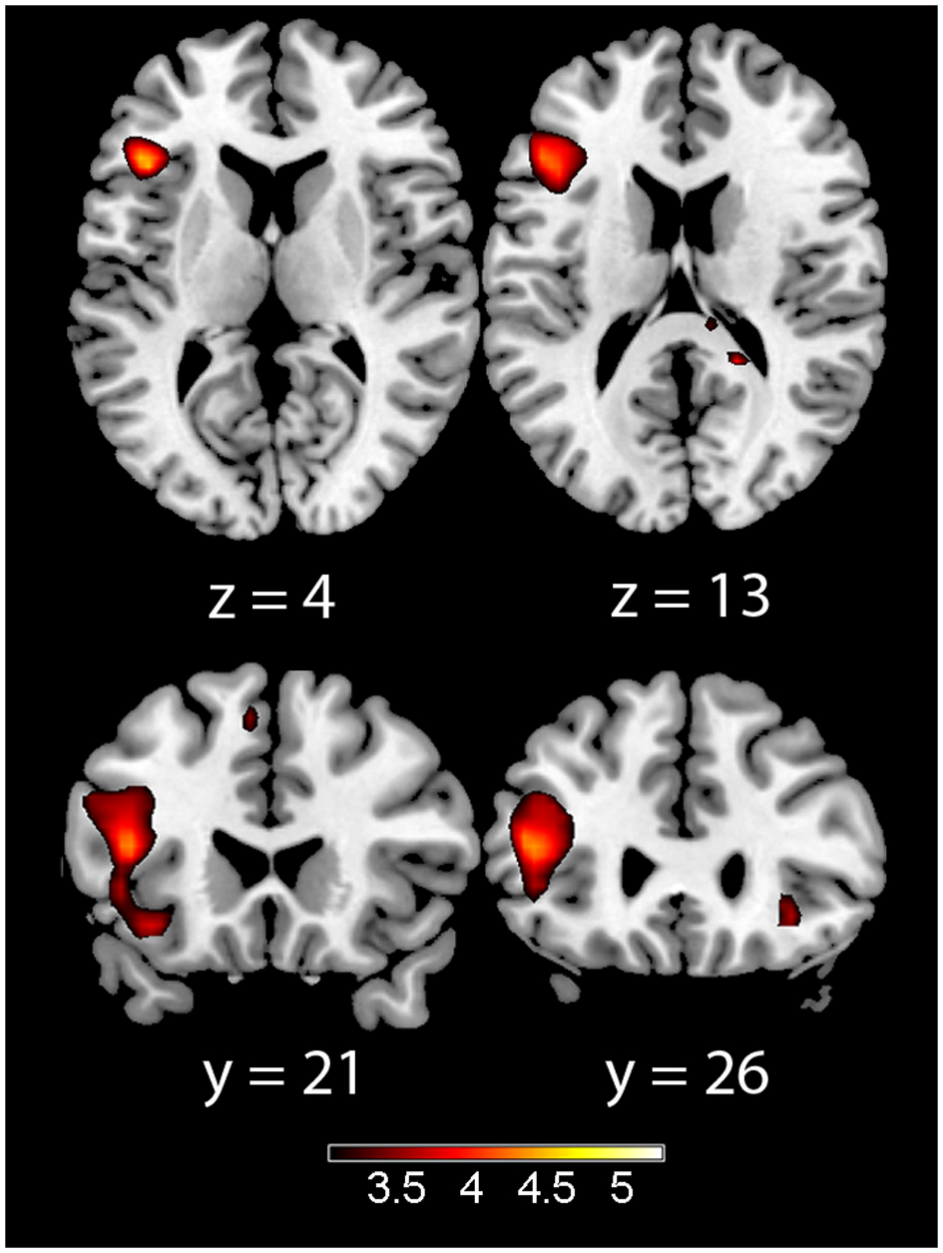
**Acquisition phase trials (early and late acquisition combined) for the incongruent > congruent contrast**. Showing left insula (left) and left inferior frontal gyrus pars trianglaris (right) on axial **(top)** and coronal **(bottom)** slices, Color map represents *t*-values.

**Figure 2:**
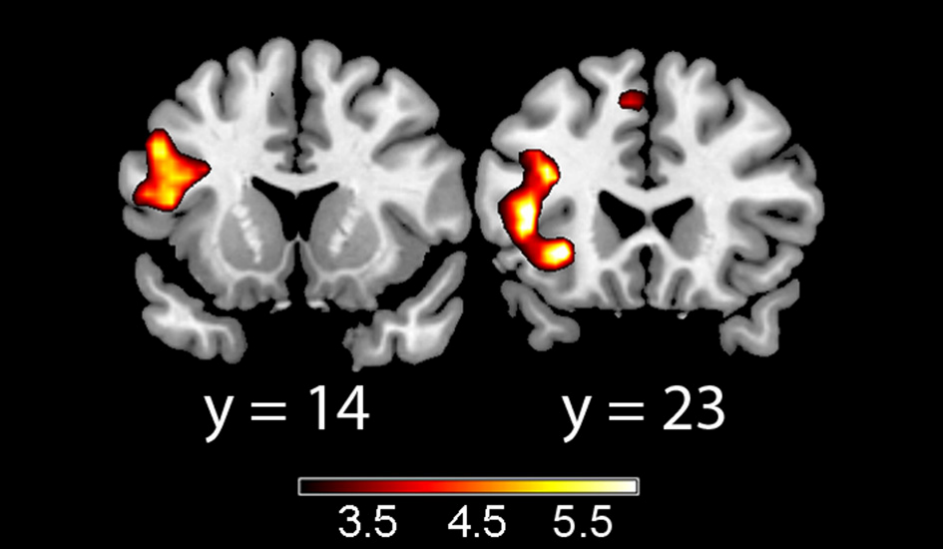
**Late acquisition phase unpaired trials for the incongruent > congruent contrast**. Showing left inferior frontal gyrus pars trianglaris (left) and left insula (right). Color map represents *t*-values.

In the acquisition phase (Table 3), significant differential activation for the incongruent vs. congruent contrast (CS1>CS2) was found in the left IFT, adjacent to the insula (Figure 1). Differential activation in the left insula was found for the incongruent vs. congruent (CS1>CS2) contrast in the early acquisition, as well as in the late acquisition, for the word-pairs that were no more followed by emotional stimuli (Figure 2).

**Table 3:** **Acquisition**.

**Contrast**	**Region**		***t*-value**	**MNI coordinate**		
				***x***	***y***	***z***
**ACQUISITION**						
Congruent > Incongruent	No differential activations					
Incongruent > Congruent	Inferior frontal triangularis	L	4.57	−45	26	13
**EARLY ACQUISITION**						
Congruent > Incongruent	No differential activations					
Incongruent > Congruent	Insula	L	4.80	−36	17	−14
**LATE ACQUISITION**						
Congruent > Incongruent	No differential activations					
Incongruent > Congruent	Insula	L	7.77	−30	23	−5

*P < 0.05 FWE corrected on cluster level; R, Right; L, Left*.

In the extinction phase (Table 4), the only differential activation was found for the congruent vs. incongruent contrast (CS2>CS1) in the anterior cingulate cortex (ACC).

**Table 4:** **Extinction**.

**Contrast**	**Region**		***t*-value**	**MNI coordinate**		
				***x***	***y***	***z***
Congruent > Incongruent without US	Anterior cingulate cortex	R	8.49	6	50	10
Incongruent > Congruent without US	No sign. Differential activations					

*P < 0.05 FWE cluster < 0.05; R, Right; L, Left*.

Finally, paired *t*-tests were computed to directly compare the incongruent vs. congruent contrast pairs in the habituation and in the extinction, as well as in the habituation and in the acquisition, not showing significant differences for corrected *p*-values.

## Discussion

The present study assessed the possibility to apply classical conditioning to two different responses (affirmative and negative) using positive and negative emotional US, in order to discriminate them. This was the first attempt to use such a protocol with fMRI. Our main hypothesis was that the effect of classical conditioning would have emerged as a differential activation for the incongruent and congruent word-pairs in emotion related areas. In fact, our results showed an effect of the aversive semantic classical conditioning, as demonstrated by the differential activation of the insula, known to be related to emotion processing (Phillips et al., 1998; Phan et al., 2002; Radua et al., 2010; Sitaram et al., 2011), and of a region adjacent to the insula, the IFT, for the incongruent vs. congruent word-pairs during acquisition. This is supported by the observation that there was no significant difference in the activation of the insula between the word-pairs in the habituation phase. The differential activation of the insula and of the IFT could be interpreted as a conditioned brain response for the incongruent word-pairs associated to the unpleasant sound. The IFT, corresponding to the BA 45 area, belongs to the inferior frontal articulatory network and is involved in phonological and semantic processing of language (McDermott et al., 2003; Amunts et al., 2004; Gold et al., 2005). More specifically, several studies have associated this area to verbal fluency (Abrahams et al., 2003) lexical search (Fiebach et al., 2002; Heim et al., 2005), and semantic memory retrieval (Rugg et al., 1999; Düzel et al., 2001). Most interestingly for our aim of developing a communication system, this region was also associated with the process of internal word generation (Friedman et al., 1998; Tremblay and Gracco, 2006). We may therefore speculate that IFT activation for the incongruent vs. congruent contrast could be related to the difference in the patterns of activation within this region pertaining “yes” and “no” responses. Hence, we propose that a multivariate pattern classifier that can discriminate between fMRI spatio-temporal patterns can be trained to decode “yes” and “no” answers for the purpose of communication.

To be able to use the present paradigm for communication, it is necessary to be able to discriminate incongruent and congruent responses also in the extinction phase. The fact that the conditioned response was evident in the late acquisition phase (when the word-pairs were not followed by emotional stimuli anymore), but not in the extinction phase, may indicate that the extinction was rapid, suggesting the need of increasing the number of trials during the acquisition phase and modifying the reinforcement schedule to establish sustained CR's. The number of acquisition trials was limited in this study, because the conditioning process took place in the fMRI scanner and we wanted to avoid tiring participants. In fact, once ascertained that the conditioning effect is visible in the acquisition phase, the first blocks of the protocol could be performed outside the scanner, and only the unpaired word-pairs would be subject to fMRI. After successful learning, the CR could be renewed faster and subsequently used for communication.

The comparisons between extinction and habituation, and between acquisition and habituation, concerning the incongruent vs. congruent contrast, are certainly important to evaluate the effect of conditioning. Although the present paradigm did not show significant differentiation for these phases, for binary communication aims, the decisive factor is the differentiation between two responses (i.e., “yes” and “no” answers). In fact, we could show that in the late acquisition phase, for the trials in which the word-pairs were not followed my emotional stimuli, there was a significant difference between incongruent and congruent word-pairs. Such differential activation could not be found in the habituation phase.

One possibility for the lack of strong differential activation for the congruent vs. incongruent word-pairs is that the appetitive US (baby-laugh) may have not been pleasant enough, although this explanation could be ruled out by considering the subjects' SAM ratings. It is possible that the presence of two distinct emotional US is confounding, so that the activations that would usually emerge with single conditioning are not elicited in double conditioning, in agreement with Lachnit (1991) and with Watt and Honey (1997). Another possible explanation for our results could be that appetitive conditioning is weaker compared to aversive conditioning, leading to weaker activations.

The lack of an independent parameter of the conditioning process, such as skin conductance response (SCR), startle response or contingency ratings, makes it difficult to judge whether the association between CS and US was identified, especially investigating a double conditioning. Nevertheless, the activations we found during acquisition, especially in the latest stage, suggests a conditioning effect for the incongruent word-pairs.

Improvements for this study comprise the reversed CS-US combination and the usage of different emotional US. To judge whether the conditioning of both CS was successful, the reversed CS-US combination should be tested, associating the congruent word-pairs with the scream and the incongruent word-pairs with the laughter, to investigate whether this results in similar CRs. Concerning a different US, Metereau et al. (Metereau and Dreher, 2012) found that both appetitive and aversive reinforcers activate the ACC, anterior insula and striatum, suggesting that the usage of appetitive and aversive US could activate the same areas, which may not be ideal for discriminating between the two conditioned responses. In our study the MCG, IFT, and SFG were activated more for the scream compared to the laughter, indicating a differentiable brain pattern but future studies should take possible overlap into consideration. It is well known that US intensity is a critical factor in effective classical conditioning. A more intense US improves learning and results in increased conditioned responses. Our suggestion for a different US would be to replace the laughter with an auditory stimulus eliciting disgust. Sitaram et al. (2011) showed that “disgust” and “sad” imagery are differentiable, indicating the possibility to classify the CRs and enable further investigation of double conditioning. Another possibility for the positive emotional US would be to use other appetitive US, such as smell or taste, depending on the individual salience of the stimuli.

The possibility to classify differential activations would be a first step in the direction of developing clinical applications, so that emotional stimuli may become tools for the conditioning of desirable behavior or brain responses. The demonstration that the response to congruent and incongruent word-pairs are differentiable (in the insula and in the IFT), at least until the late acquisition phase, introduces the possibility of applying such a paradigm for practical purposes, such as a binary BCI, which could allow basic “yes/no” communication with patients suffering from cognitive impairment.

Another aspect that should be considered when applying this classical conditioning protocol for basic communication is subject variability. Some individuals may be more easily conditioned, while others may require a higher number of trials in which word-pairs are associated to emotional stimuli. This introduces the need of developing an “adapted acquisition” protocol, based on the subjective ease of conditioning. For instance, if specialized pulse sequences become more sensitive to amygdala activation while enabling real-time fMRI for pattern classification, or if other biomarkers of acquisition are identified, it would be possible to obtain extra information about the conditioning process, both to improve the paradigm in the future, and to adapt it to different individuals.

## Conclusion

This study represents a first step into a new direction of double conditioning of brain responses. The insula activations for unpaired word-pairs during the late acquisition suggest that conditioning took place for the incongruent word-pairs. The importance of the insula and neighboring inferior frontal gyrus pars triangularis throughout the association process is shown by the continuous differential activation during the different phases of conditioning. The possibility of using classical conditioning with emotional US opens the door to investigating more variations on double conditioning with different US or testing the usability for clinical applications.

The application we aimed at is a BCI for communication. If the negative and affirmative response can be classified from the BOLD response, this possibility could become reality (i.e., allowing to discriminate between affirmative and negative thinking). This application would be most beneficial for completely locked-in and cognitively impaired patients.

### Conflict of interest statement

The authors declare that the research was conducted in the absence of any commercial or financial relationships that could be construed as a potential conflict of interest.
